# Diversity, Ecology and Phytogeography of Bryophytes across Temperate Forest Communities—Insight from Mt. Papuk (Croatia, SE Europe)

**DOI:** 10.3390/plants12193346

**Published:** 2023-09-22

**Authors:** Antun Alegro, Vedran Šegota, Anja Rimac, Beáta Papp

**Affiliations:** 1 Division of Botany, Department of Biology, Faculty of Science, University of Zagreb, Marulićev trg 20/II, 10000 Zagreb, Croatia; antun.alegro@biol.pmf.hr (A.A.); anja.rimac@biol.pmf.hr (A.R.); 2 Botanical Department, Hungarian Natural History Museum, Pf 222, H-1476 Budapest, Hungary; papp.beata@nhmus.hu

**Keywords:** biodiversity, biogeography, Ellenberg’s indicator values, habitats, liverworts, mosses, SE Europe

## Abstract

It has been widely documented that the complex structure of forest ecosystems supports considerable bryophyte species and functional diversity. In this study, we assessed the diversity, distribution and ecological and phytogeographical features of bryophytes across a gradient of temperate forest types on Mt. Papuk. This is the largest and highest mountain in the lowland, Pannonian part of Croatia, with high geological diversity and various temperate forests covering 95% of the mountain. According to the predominant tree species (oak vs. beech), geological bedrock (calcareous vs. siliceous) and soil reaction (alkaline vs. acidic), 21 study plots were classified into four distinct forest types. In all, 184 bryophyte species (35 liverworts and 149 mosses) were recorded. Although the forest types investigated did not differ significantly with respect to species richness, each was characterized by a considerable number of diagnostic bryophyte species. According to our results, one of the main ecological factors determining the variability of the forest bryophyte composition was geological bedrock and the associated soil reaction. Basiphilous forests developed on carbonate bedrock harbored more thermophilous and nitrophilous bryophytes and were characterized by southern-temperate and Mediterranean–Atlantic biogeographic elements. In contrast, acidophilous forests growing on silicate bedrock were characterized by wide-boreal and boreo-arctic–montane elements, i.e., bryophytes indicating cooler habitats and nitrogen-deficient soils. Based on the results, we hypothesized that the main latitudinal biogeographic distinction between southern and northern biogeographic elements is driven more by geological substrate than by the main tree species in forest communities. The present study confirmed previous findings that bryophytes are good and specific habitat indicators and show associations with different forest types, which can help to understand the complexity, ecological microconditions and biogeographic characteristics of forest communities.

## 1. Introduction

Bryophytes are a significant component in forest ecosystems, which, due to their complex structure, provide many different substrates and niches supporting considerable bryophyte species richness and functional diversity [1,2,3,4,5,6]. Bryophytes serve several important functions in the forest ecosystem. They are colonists of bare soil and they are capable of absorbing moisture quickly, retaining it and releasing it slowly, acting as a water reservoir [7]. In temperate forests, their water storage capacity prevents the rainwater run-off and ensures that nutrients from the rainwater are fed into the ecosystem [8]. Furthermore, the bryophytes contribute significantly to the overall energy flow and nutrient cycling in the woodland environment [7]. For example, mosses represent 49% of the annual phytomass production, and they contribute 75% to the phosphorus supply of the forest ecosystem [8]. However, they provide a habitat for a huge number of invertebrates, fungi and other microorganisms [7]. Furthermore, mosses that form mat-like colonies help to prevent soil erosion, and they create seed beds influencing tree growth by either enhancing or prohibiting seedling survival [9,10]. 

The bryophyte species composition and richness in forest ecosystems are primarily driven by macroclimatic factors such as air temperature and precipitation [11], longitudinal and altitudinal gradients [12,13], microrelief diversity [14] and forest integrity [15], as well as forest management and silviculture practices [16,17,18,19]. On a smaller scale, the microclimatic conditions (shade, humidity), substrate availability and light conditions determined by forest structure significantly affect bryophyte diversity and cover [6,20,21,22,23,24]. In addition, soil properties, especially soil reaction, also affect forest floor bryophyte assemblages and their diversity [25,26,27,28]. Finally, anthropogenic impacts such as habitat disturbance caused by logging can have a considerable influence on forest bryophyte diversity [29,30]. 

In temperate and boreal forests, vascular plants and forest floor bryophytes are major components of understory vegetation [31]. Both groups are often considered equally important components of plant communities and are thus both included in the vegetation relevés and used as diagnostic species of the same community types [32]. According to several authors [33,34,35], bryophyte composition is significantly correlated to different environmental conditions, making bryophytes reliable indicators of forest condition and naturalness [19,36]. Ewald [31,37] showed that bryophyte composition in mountain forests is significantly correlated to environmental conditions, but less so than vascular plants. Although Diekmann [33] assumed that this limited indication potential of bryophytes is a consequence of their low species numbers in deciduous forests, Ewald [31] showed that the higher indication potential of vascular plants is not principally due to their higher species diversity but can be attributed to a more pronounced response of vascular plants to ecological gradients. Furthermore, he concluded that forest floor bryophytes do not contribute substantially to bioindication in terms of Ellenberg’s indicator values in mountain forests.

Nevertheless, plenty of studies have shown that bryophytes have high indication potential. They can be considered as good indicators of environmental conditions in natural forest communities [38] and promising indicators of forest integrity [15]. The richness and composition of bryophytes have a high indication value for conservation strategies of set-aside forests [39] and bryophyte community composition is known to indicate fragmentation in temperate rainforests [40]. It was shown on a large data set that, in Central Europe (Poland), bryophytes are good indicators of habitat conditions and show associations with different types of forests [41]. Similar patterns were obtained for Slovenian forests; it was demonstrated that, in close-to-nature managed forests, bryophyte species composition varied considerably among five forest types of different tree layer composition (broadleaved vs. conifers) and bedrock and soil type (calcareous vs. siliceous) [42]. Similarly, bryophyte species richness and species composition in Alpine forests were best explained by substrate and forest type [4]. In addition, it was elaborated that tree species composition (deciduous vs. coniferous) determines bryophyte species richness and cover and that soils with lower pH and macronutrient content support larger bryophyte cover [43]. However, it was demonstrated that the stand structure of temperate deciduous forests was more influential on forest floor bryophyte diversity than soil characteristics [44]. 

However, to date, there has been no research on bryophyte diversity nor their ecological and phytogeographical features across forest communities in Croatia. Moreover, Croatia is bryologically still quite underexplored. Therefore, the aim of this work was to obtain a detailed insight into the diversity of the bryophyte flora of Mt. Papuk. Since this area harbors considerable geological and vegetational diversity, we further wanted to explore the phytogeographical, coenological and ecological structure of bryophyte flora in relation to different temperate forest communities in order to assess the indication potential of forest bryophytes. 

## 2. Results

In all, 184 bryophyte species—35 liverworts and 149 mosses—were recorded within the forest communities studied (Table A1 and Table S1). The most frequent species across the sampling plots was *Hypnum cupressiforme* (81%), followed by *Polytrichum formosum* (52%), *Isothecium alopecuroides* (48%), *Dicranella heteromalla* (48%), *Brachythecium rutabulum* (48%), *Plagiochila porelloides* (48%) and *Metzgeria furcata* (43%). Nevertheless, as many as 74 species appeared on only one sampling plot. Noteworthily, several species recorded, such as *Microlejeunea ulicina*, *Rhabdoweisia fugax*, *Sciuro-hypnum flotowianum*, *Dicranum spurium* and *Syntrichia calcicola*, are known in Croatia only from Mt. Papuk. Furthermore, species listed in Annex V of the Habitats Directive [45], *Leucobryum glaucum* and *Sphagnum quinquefarium*, were recorded as well, with the population of *S. quinquefarium* from Papuk being the largest known in Croatia.

Diagnostic species (Table A1) for acidophilous beech forests (FA) were *Isothecium alopecuroides*, *Plagiochila porelloides*, *Metzgeria furcata*, *Rhizomnium punctatum* and *Lophocolea heterophylla* and diagnostic species for basiphilous beech forests (FB) were *Mnium stellare*, *Plagiomnium rostratum*, *P. undulatum*, *Brachythecium tommasinii*, *Conocephalum salebrosum* and *Homalothecium philippeanum*. Diagnostic species for acidophilous oak forests (QA) were *Polytrichum piliferum* and *Ceratodon purpureus*, while the basiphilous oak forests (QB) had a larger set of diagnostic species comprising *Leucodon sciuroides*, *Barbula unguiculata*, *Tortula muralis*, *Encalypta streptocarpa*, *Homalothecium lutescens*, *H. sericeum*, *Didymodon acutus*, *Grimmia pulvinata*, *Tortella tortuosa* and *Flexitrichum flexicaule*.

**Figure 1:**
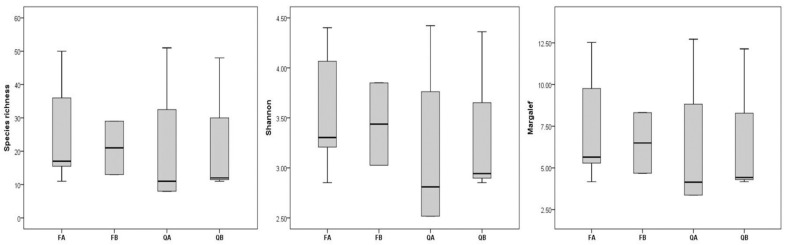
Bryophyte alpha diversity indices across investigated forest types. FA—acidophilous beech forests, FB—basiphilous beech forests, QA—acidophilous oak forests, QB—basiphilous oak forests.

Although the distinction between forest types based on bryophyte diversity (species richness, Shannon diversity index and Margalef’s richness index) was not supported by the Kruskal–Wallis test, or by the Mann–Whitney pairwise test (Table S2), the beech forests exhibited higher diversity indices than oak forests (Figure 1). However, considering the species number, the richest site, with 51 species, was within an acidophilous oak forest, followed by two sites in acidophilous beech forests (50 species each) and by one site in a basiphilous beech forest (48 species) (Table S1). It was the complex structure of microhabitats observed in these sites (rocks, rock crevices and peat deposits) that ensured such a high number of species. 

**Figure 2:**
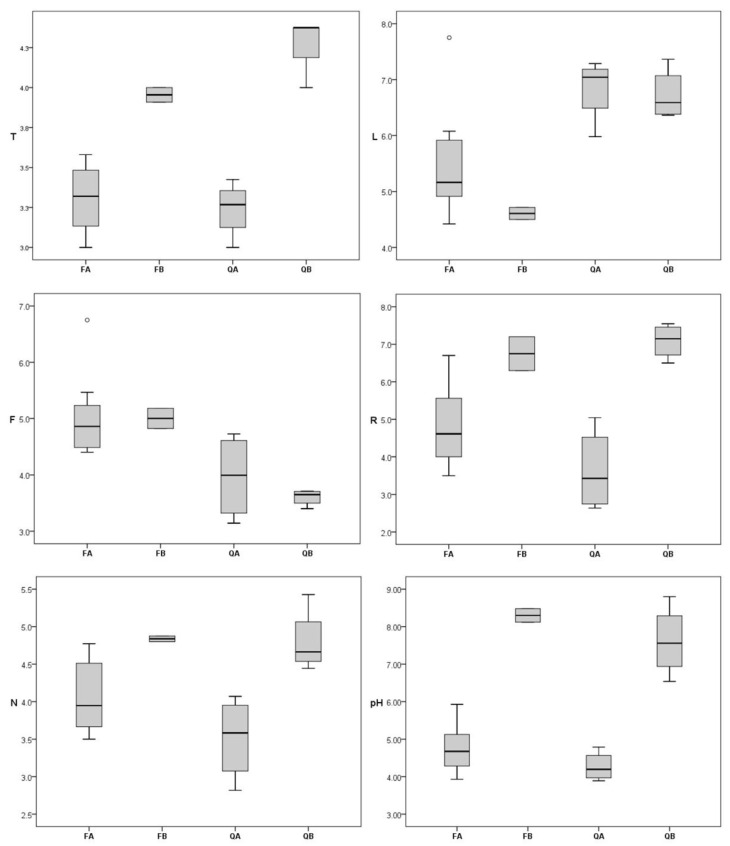
Box-plot graphs of bryophyte Ellenberg’s indicator values (EIVs) for temperature (T), light (L), moisture (F), reaction (R), nutrients (N) and measured soil pH across investigated forest types. FA—acidophilous beech forests, FB—basiphilous beech forests, QA—acidophilous oak forests, QB—basiphilous oak forests.

Ellenberg’s indicator values of bryophyte flora (EIVs) and measured pH (Figure 2) values showed clear differences between the studied forest types, which were supported by the Kruskal–Wallis test (*p* < 0.05 in all cases). The subsequent Mann–Whitney pairwise test revealed the differences between particular forest types (Table S2). Both groups of oak forests (QA and QB) were characterized by higher light values (L), i.e., by more heliophytic bryophyte flora, and by lower moisture values (F) indicating drier habitats, with QB being the driest and significantly different from all other types. Semi-shade species of moderately moist to moist soils were specific for beech forests, while species of well-lit places (occurring also in partial shade) and well-drained soils were characteristic for oak forests. Both basiphilous forest types (FB and QB) had significantly higher pH values than beech forests, which were supported by higher bryophyte EIVs for soil reaction (R). Furthermore, oak forests showed higher bryophyte EIVs for temperature (T) and nutrient content (N). 

**Figure 3:**
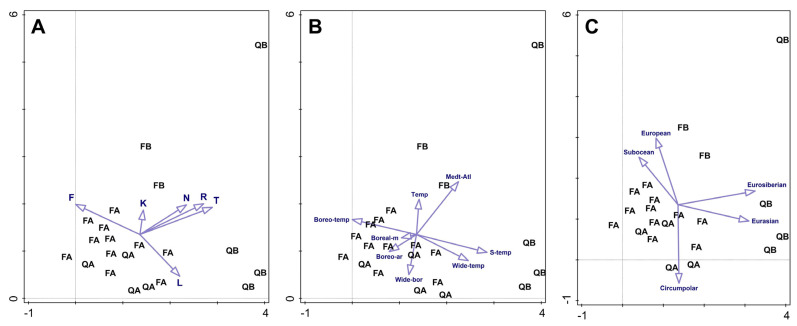
DCA ordination graphs of investigated plots in different forest types with Ellenberg’s indicator values (**A**), biogeographic elements based on major biome (**B**) and eastern limit (**C**) passively projected as vectors. FA—acidophilous beech forests, FB—basiphilous beech forests, QA—acidophilous oak forests, QB—basiphilous oak forests; Ellenberg’s indicator values: T—temperature, L—light, F—moisture, R—reaction, N—nutrients; biogeographic elements: Temp–temperate, Boreo-temp—oreo-temperate, Boreal-m—boreal–montane, Boreo-ar—boreo-arctic–montane, Wide-bor—wide-boreal, Wide-temp—wide-temperate, S-temp—southern-temperate, Med-Atl—Mediterranean–Atlantic.

All these tendencies were furthermore corroborated by DCA analysis (Figure 3A). Furthermore, acidophilous beech forests (FA) were grouped mainly along the moisture vector (F), while basiphilous oak forests (QB) were the most distant from other forest types and grouped in the directions of vectors for temperature (T), soil reaction (R), nutrients (N) and light (L). Additionally, acidophilous oak forests (QA) were separated from the acidophilous beech forests (FA) along the light vector.

The DCA graph representing the relationship between sampling plots and the bryophyte major biome elements (Figure 3B) indicates that basiphilous and acidophilous forests were biogeographically clearly distinguished. The basiphilous ones were characterized by different temperate elements, as well as Mediterranean–Atlantic bryophyte elements, whereas acidophilous forests were characterized by boreal bryophyte elements.

**Figure 4:**
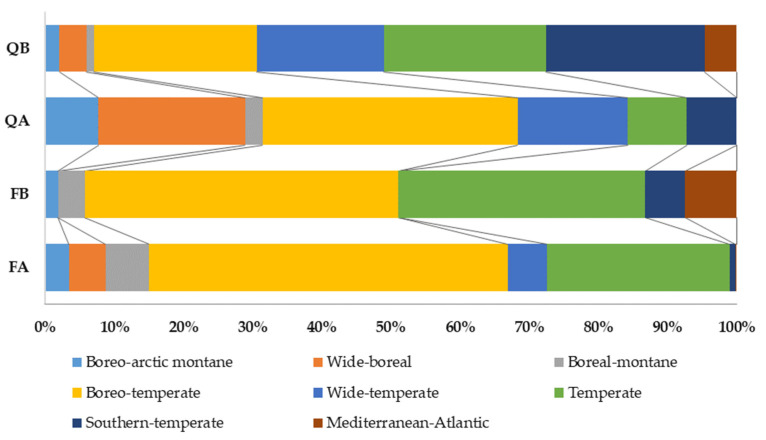
Spectrum of biogeographic elements regarding major biomes (E1) for the bryophyte flora of Mt. Papuk through forest types. FA—acidophilous beech forests, FB—basiphilous beech forests, QA—acidophilous oak forests, QB—basiphilous oak forests.

The bryophyte biogeographic spectrum regarding distribution throughout the major biomes (E1) (Figure 4) showed that the dominant element in all forest types was boreo-temperate, but with the highest ratio in acidophilous beech forests (51.8%). The share of boreo-arctic–montane and wide-boreal elements was the highest in acidophilous oak forests (7.7 and 21.3%, respectively). By contrast, the highest shares of southern-temperate (22.9%) and wide-temperate elements (18.4%) were in basiphilous oak forests, while the highest shares of temperate (35.7%) and Mediterranean–Atlantic species (7.5%) were characteristic of basiphilous beech forests. The Mediterranean–Atlantic element was absent in both acidophilous forest types, whereas the southern-temperate element was represented with only 0.8% in acidophilous beech forests. Generally, oak had more complex spectra of bryophyte biogeographical elements than beech forests.

In the DCA graph (Figure 3C) showing the relationship of the forest sampling plots and the bryophyte eastern limit (E2), basiphilous oak forests are shifted toward the vectors representing Eurosiberian and Eurasian elements, and basiphilous beech forests toward European and suboceanic elements. The latter element also characterized acidophilous beech forests, while acidophilous oak forests were shifted toward the vector representing a circumpolar element.

**Figure 5:**
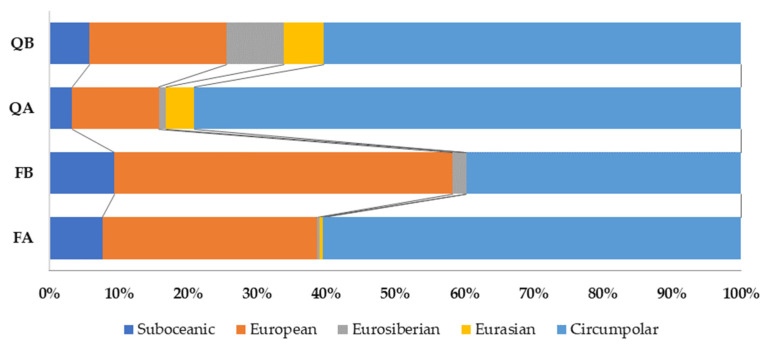
Spectrum of biogeographic elements regarding eastern limit category (E2) for the bryophyte flora of Mt. Papuk through forest types.

When the spectrum of the eastern limit category of bryophyte species distribution was considered (Figure 5), the circumpolar element was dominant in all forest types, with the highest share in acidophilous oak forests (79.1%). An exception was found in basiphilous beech forests, where the European element (48.9%) dominated. The Eurasian element was present exclusively in the oak forests (5.8% in QB and 4.1% in QA), while, in the beech forests, it was absent (FB) or marginally present (0.57% in FA). Bryophyte species of the suboceanic element were more frequent in beech (9.4% in FB and 7.6% in FA) than in oak forests (5.8% in QB and 3.3% in QA). Finally, the Eurosiberian element had by far the highest share in basiphilous oak forests (8.3%), but was present with less than 2% in all other forest types.

The correlation between the ecology and the biogeography of forest bryophytes was demonstrated by the Mantel test. Correlations between the matrix of bryophyte Ellenberg’s indicator values and both biogeographical matrices were high (r (E1) = 0.7 and r (E2) = 0.5) and statistically significant (*p* < 0.001). A positive association between matrices was also indicated by the randomization test.

## 3. Discussion

The results of this study show that bryophyte flora corresponds with forest vegetation both ecologically and biogeographically and that the interconnection between bryophytes and forest vegetation observed on a small scale in this study demonstrates the same patterns detected on mid- [4] and large national scales [41,42]. Noteworthily, the total number of recorded bryophyte species in our study (184 spp.) corresponds closely with the overall forest bryophyte diversity of similar Slovenian (199 spp.) [42] and Polish forests (173 spp.) [41].

Diagnostic bryophyte species could be defined for all studied forest types and their number ranged in our study from 2 to 10, which is in line with the number of diagnostic species for Central European forests, which ranged from 1 to 12 [41]. However, the φ-coefficient threshold was conservatively set much higher in our study (40% vs. 15%). The presence of valid diagnostic bryophyte species, as well as their quite high number in our study, corroborates previous findings on the relationship between bryophyte assemblages and forest communities [4,35,42,46,47,48]. By contrast, Stefańska-Krzaczek et al. [41] found a low number of diagnostic bryophyte species, suggesting their subordinate position compared to vascular plants in vegetation classification, as already noted by some authors [31,46]. However, many forest communities, especially those that are acidophilous, contain a low number of diagnostic vascular plant species and, in these cases, bryophytes can substantially contribute to the vegetation classification [49,50]. 

Although in our study the species richness did not differ significantly among investigated forest types, a pattern in which beech forests have both significantly higher bryophyte EIVs for moisture (F) and also higher diversity indices than oak forests was evident. This is consistent with the findings of Stefańska-Krzaczek et al. [41] that the moisture index is the most important predictor of the number of forest bryophyte species. 

One of the main ecological factors determining the variability of the forest bryophyte flora is soil reaction [25,26,27,28]. According to our results, basiphilous forest communities, either beech or oak, include more thermophilous and nitrophilous bryophyte species, which can be explained by more aerated and therefore warmer soil due to a porous and fragmented carbonate or dolomite bedrock. Good aeration and non-acidic soil reaction enhance the mineralization of organic matter, which in turn makes these habitats suitable for colonization by more nitrophilous species. In terms of biogeography, these basiphilous forests were characterized by southern-temperate (*Frullania dilatata*, *Didymodon acutus*, *D. fallax*, *Gymnostomum calcareum*, *Grimmia pulvinata*, *Homalothecium lutescens*, *H. sericeum*, *Trichostomum crispulum*, *Syntrichia calcicola*, *Tortula muralis*) and Mediterranean–Atlantic biogeographic elements (*Plasteurhynchium striatulum*, *Tortella squarrosa*, *Didymodon sinuosus*, *Rhynchostegiella teneriffae* and *Trichostomum brachydontium*). However, acidophilous forest communities were characterized by wide-boreal (*Jungermannia pumila*, *Dicranum scoparium*, *Hylocomium splendens*, *Polytrichum commune*, *P. juniperinum*) and boreo-arctic–montane elements (*Blepharostoma trichophyllum*, *Cynodontium polycarpon*, *Dichodontium pellucidum*, *Pogonatum urnigerum*), i.e., bryophytes indicating cooler habitats and nitrogen-deficient soils. Acidic substrates are mostly siliceous, nonporous and less aerated. This implies a relatively cool soil in comparison to the carbonate and dolomite soils in the same region, and a slower mineralization of organic matter, not only due to the cooler conditions but, and primarily, because of the acidic soil reaction. This, therefore, may suggest that the main latitudinal biogeographic distinction between southern and northern biogeographic elements is driven by geological substrate and not by the main tree species in forest communities.

The distinction between longitudinal eastern and western biogeographic elements is observable in the oak–beech dichotomy. While the oak forests feature bryophyte flora with higher indicator values for light and lower values for moisture, the opposite is true for the beech forests. This may be explained by the density of forest stands and the relief of their habitats. Namely, in our study, which included zonal temperate forest, oak forest stands were, as expected, less dense than beech. This is partially a consequence of looser canopy closure and partially due to the relatively thin soil layer on often steep and inclined positions on which the oak stands investigated are developed [49]. For these reasons, water is a limiting factor, preventing the development of dense forest stands, which in turn drives the colonization of light-demanding bryophyte species of well-drained soils. The presence of Eurasian and Eurosiberian species (e.g., *Brachythecium glareosum*, *Homomallium incurvatum*, *Homalothecium sericeum*, *Leucodon sciuroides*) almost exclusively within the oak forests investigated reflects a continental, more “eastern” climate with a pronounced dry summer period and a high annual temperature amplitude. On the other hand, the European (e.g., *Ctenidium molluscum*, *Plagiomnium rostratum*, *P. undullatum*, *Thamnobryum alopecurum*) and suboceanic (*Bazzania trilobata, Calypogeia fissa, Diplophyllum albicans, Atrichum angustatum, Heterocladium heteropterum, Pseudotaxiphyllum elegenas*, etc.) species were the most abundant in the beech forests. Such a biogeographic spectrum makes the beech forests more “western” than the oak forests, indicating microclimatic conditions with more constant humidity and temperature.

The present study confirmed the previous findings that bryophytes are good and specific habitat indicators and that they show a clear association with different forest types [4,41,42,43], which is useful for the understanding of the complexity, ecological microconditions and biogeographic characteristics of the forest communities. It is important to note that non-vascular plants are rarely included in floristic and vegetation assessments since their importance as indicators of the plant communities is still not truly recognized [51]. Consequently, there has been little systematic study of their biogeography and community ecology [52]. Ewald [31], based on research into mountain forests in the Bavarian Alps, concluded that combining vascular plants and bryophytes yielded very similar or even slightly less stringent relationships with the environment than using vascular plants only. Moreover, he showed that bryophyte-based indicator values do not significantly predict the residuals of measured ecological variables against vascular-plant-based Ellenberg indicator values. On the other hand, Bagella [52], from a review of twenty-seven papers, concluded that vascular plants cannot be used as habitat indicator surrogates for bryophytes without carefully considering habitat type, environmental and human-induced factors and assessment scale. This conclusion is strongly supported by the fact that, in some cases, richness in two taxonomic groups is affected similarly by the same environmental factors, and differently by others. Several studies pointed out that the species richness of these groups responds similarly to precipitation [53] and moisture [54], but differently to pH, conductivity and water level dynamics [55].

The results of this study can improve forest conservation strategies and management plans. Ecological and biogeographical analysis of bryophyte flora has shown that acidophilic beech forests, especially moist ones, stand out with a high proportion of boreal and boreal–arctic–montane species, which are rare in Croatian flora due to its geographical position. Protection of their habitats is one of the priorities while preserving the peculiarities of bryophyte flora and generally the biodiversity in Croatia. In addition, some of these habitats are unique in Croatia, e.g., beech forests with dominating peat moss *Sphagnum qiunquefarium* (plot 18 in Table S1), which is also the only place in Croatia where *Dicranum spurium* and some other rare bryophytes are found. It is worth noting that some of them (e.g., *Bazzania trilobata*, *Calypogeia fissa*, *Diplophyllum albicans*, *Pseudotaxiphyllum elegans*) belong to the rare subocanic element. Such forest habitats with permanent soil moisture enabling the survival of northern and oceanic elements should be exempted from any timbering and coppicing. Moreover, they are the most threatened by climate change due to their wetter and mostly cooler microclimate in comparison to the general climate of the area. The analyses of climatic data have shown that changes in the temperature and precipitation regime have already begun in Croatia [56]. In eastern Croatia, where the study area is situated, a considerable increase in the temperature of the coldest and the warmest months is expected, as well as a decrease in the precipitation in the driest and the wettest months [56]. On the other hand, oak forests harbor a high proportion of thermophilous and light-demanding species belonging to the temperate and southern temperate elements. These forests often form a mosaic landscape with former extensive dry pastures. Abandonment of these pastures and their encroachment into thickets also affect the forests due to the spread of the successional species into the forests, which could change forest structure, light conditions and microclimate. Therefore, to preserve these forests in the mosaic landscape and their specific biodiversity, it is necessary to prevent the succession of grasslands. These oak forests are, according to the models, also susceptible to climate change, which can alter their spatial distribution and cause their possible disappearance and substitution with other forest types [56].

The use of bryophytes as indicators is strongly supported by our results, as they broaden the understanding of the complexity, biogeography and environmental conditions of plant communities.

Our research was restricted to only one mountain in the Peripannonian area. Future research, including the broader area and neighboring mountains, will give a more complete insight into the diversity and ecology of forest bryophytes in that unique area that connects two biogeographical regions, Pannonian and Dinaric.

## 4. Materials and Methods

### 4.1. Study Area

**Figure 6:**
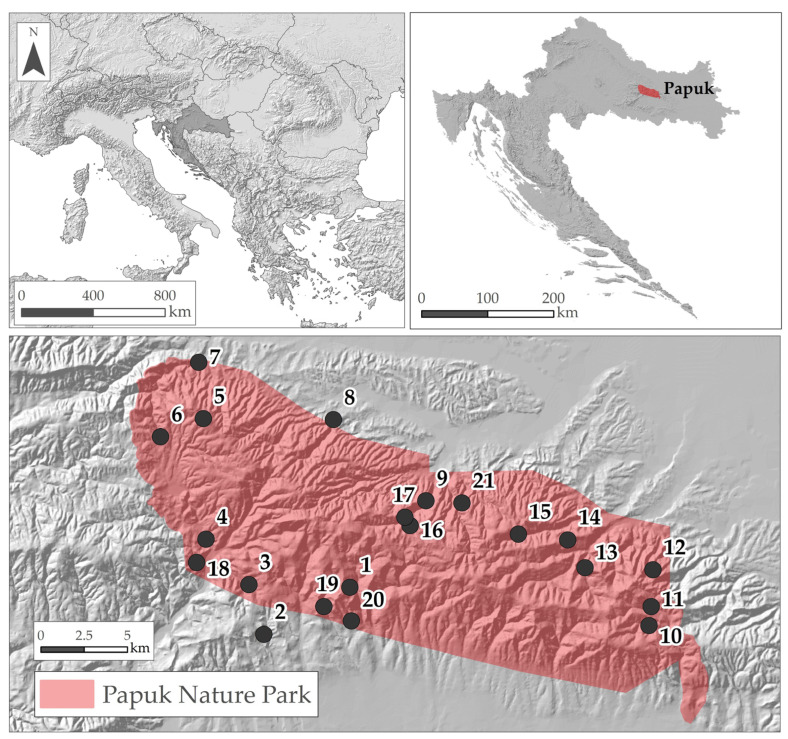
Geographical position of the study area in southeast Europe and Croatia and distribution of the investigated plots on Mt. Papuk.

The field survey of the forest bryophytes was conducted on Mt. Papuk, which is situated in the eastern part of Croatia, on the southern border of the large Pannonian plain, within the Peripannonian biogeographic region characterized by a wetter climate than that of the Pannonian region (Figure 6). In this region, Papuk is the largest and highest mountain; the peaks reach between 800 and 953 m a.s.l. Unlike the majority of Croatian mountains, which are built of Mesozoic limestone, Papuk has a high geological diversity dominated by metamorphic rocks, granites and different types of schists. The climate is temperate but moderately warm without an explicit dry period. Depending on the elevation, the annual mean temperature is between 8 and 11 °C and annual precipitation varies between 800 and 1300 mm. Ninety-five per cent of the area is covered with beech forests with dominant acidophilous beech communities of the suballiance *Luzulo luzuloidis-Fagenion* (Lohm. et Tx. 1954) Oberd. 1957 and the beech–fir community (ass. *Festuco drymeiae-Abietetum* Vukelić et Baričević 2007). The basiphilous beach forests are less frequent, belonging to ass. *Vicio oroboidi-Fagetum sylvaticae* (Horvat 1938) Pocs et Borhidi in Borhidi 1960 and developed above patches of carbonate and dolomite rocks. Similarly, oak forests are also either acidophilous (several communities from the alliance *Quercion robori-petraeae* Br.-Bl. 1932) or basiphilous (*Quercion pubescenti petraeae* Br.-Bl. 1932) [49,57]. Mt. Papuk, with an area of 33,600 ha, has been protected as a Nature Park since 1999, and recently as a NATURA 2000 site [58].

### 4.2. Methods

**Figure 7:**
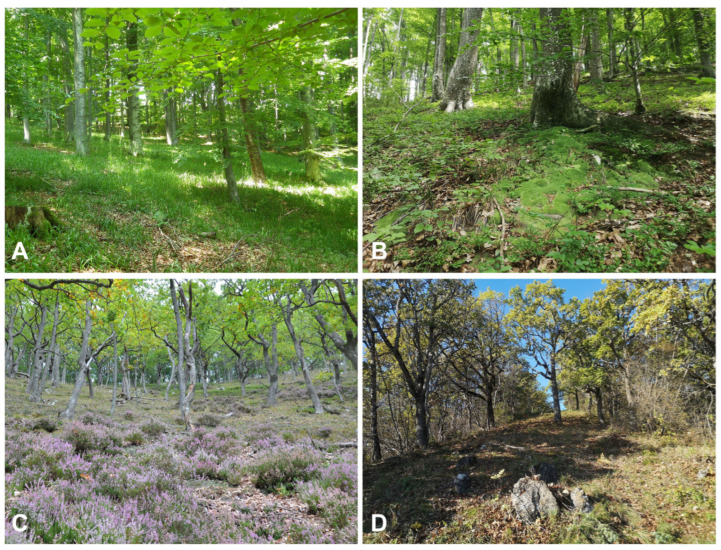
Investigated forest types. (**A**)—acidophilous beech forest (FA); (**B**)—basiphilous beech forest (FB); (**C**)—acidophilous oak forests (QA); (**D**)—basiphilous oak forests (QB).

Forest bryophytes were collected during a four-year period (2009–2013) from the forest floor, rocks and stones, living wood (bark of living trees) and deadwood (stumps, lying logs and standing dead trees). In total, 22 sampling plots (each 200 m^2^) were selected so that the four main forest communities present on Papuk (Figure 7 and Table A2) were represented in proportion to their frequency within the area. These types, defined by the main tree species, soil reaction and bedrock type (calcareous vs. siliceous), are:

- FA—acidophilous beech forests (suballiance *Luzulo luzuloidis-Fagenion* (Lohm. et Tx. 1954) Oberd. 1957 and ass. *Festuco drymeiae-Abietetum* Vukelić et Baričević 2007);
- FB—basiphilous beech forests (ass. *Vicio oroboidi-Fagetum sylvaticae* (Horvat 1938) Pocs et Borhidi in Borhidi 1960 from the suballiance *Epimedio-fagenion* (Borhidi 1963) Martinček et al. 1993);
- QA—acidophilous oak forests (ass. *Festuco drymeiae-Quercetum petrae* (Janković 1968) Hruška-Dell’Uomo 1975, ass. *Molinio arundinaceae-Quercetum petraeae* Šugar 1972 and *Quercus petraea-Calluna vulgaris* community, all three belonging to the alliance *Quercion robori-petraeae* Tx. (1931) 1937);
- QB—basiphilous oak forests (*Lathyro nigri-Quercetum petraeae* Horvat (1938) 1958, *Fraxino orni-Quercetum pubescentis* Klika 1938, both from the alliance *Quercion pubescenti-petraeae* Br.-Bl. 1932).

On each sampling plot, the geological bedrock was documented and a composite soil sample was collected for soil reaction (pH) measurement in suspension with distilled water according to a standard protocol [59].

The bryophyte specimens are deposited in the Herbarium of the Botanical Department of the Faculty of Science in Zagreb (ZA) and the Herbarium of the Hungarian Natural History Museum in Budapest (BP). Nomenclature of the bryophyte taxa follows Hodgetts et al. [60]. 

In the statistical analyses, bryophyte species recorded on different substrates in each plot were analyzed together.

The forest types were analyzed with the respect to species composition. For each species within a particular forest type, frequency (f) was calculated, as was the fidelity (φ) coefficient, tested for significance with the Fisher test (Table A1), using Juice 7.1 [61]. These were used to define diagnostic species (f ≥ 50%; φ ≥ 40%, *p* < 0.05).

Ellenberg’s indicator values (EIV) for light (L), moisture (F), reaction (R), nitrogen (N) and temperature (T) were used in the ecological analysis [62], while chorological analysis followed the system proposed by Hill and Preston [63], which was elaborated in Hill et al. [64] and recently summarized by van Zuijlen et al. [62]. Both biogeographic elements, reflecting the major biomes (E1) and the eastern limit in Eurasia (E2), were used. Spectra of EIVs and biogeographic elements were calculated for each sampling site and for each group of forest communities (FA, FB, QA and QB). 

Bryophyte alpha diversity indices (species richness, Shannon diversity index and Margalef’s richness index) of different forest types were calculated in Past 4.12 software [65]. Furthermore, the Kruskal–Wallis test for equal medians followed by the pairwise Mann–Whitney test were performed using the same software to compare alpha diversity indices, bryophyte EIVs and measured pH among the forest types. 

EIVs, pH values and alpha diversity indices were visualized through boxplot graphs in SPSS 22.0 software. 

Community structure was assessed using the indirect ordination method, detrended correspondence analysis (DCA). In DCA, EIVs and both biogeographical elements (E1 and E2) were passively projected over ordination of plots to assess possible environmental and biogeographical gradients. DCA revealed that the data were compositional with a gradient longer than 3.0 SD units, confirming that analysis based on a unimodal model, such as DCA, was suitable for describing the data [66]. The procedure was performed in CANOCO 5.0 [66,67].

The Mantel test, a permutation test for correlation between two distance or similarity matrices, was used to test the possible relationship between ecology (described by EIVs) and biogeography (biogeographical elements E1 and E2). The analysis was performed using the Sorensen distance measure and 999 randomized runs. The calculated R value is the Pearson correlation coefficient among all the entries in the two matrices and it was retained as significant if *p* < 0.001. The test was performed in PcOrd 7.0 [68].

## 5. Conclusions

The present study confirmed previous findings that bryophytes are good, specific habitat indicators, and show associations with different forest types. We defined diagnostic species for each forest type based on frequency and fidelity index and their number varied from two in the acidophilous oak forests to 10 in basiphilous beech forests. Two main ecological and biogeographical gradients were recognized—latitudinal and longitudinal. The main latitudinal biogeographic distinction between southern and northern biogeographic elements is driven largely by geological substrate rather than by the main tree species in forest communities. Basiphilous forests developed on carbonate bedrock harbored more thermophilous and nitrophilous bryophytes and were characterized by southern-temperate and Mediterranean–Atlantic biogeographic elements. By contrast, acidophilous forests developed on silicate bedrock were characterized by wide-boreal and boreo-arctic–montane elements, i.e. bryophytes indicating cooler habitats and nitrogen-deficient soils. Furthermore, the latitudinal distinction between eastern and western biogeographical elements was observed in the oak–beech dichotomy. While the oak forests featured bryophytes with higher indicator values for light and lower values for moisture, the opposite was true for beech forests. The presence of Eurasian and Eurosiberian species was almost exclusively limited to oak forests. On the other hand, the European and suboceanic species were the most abundant in beech forests. The findings of this study can have direct applications in conservation and forest management, suggesting a strong need for the controlled exploitation of beech stands with permanent soil moisture, as well as a need for the suppression of vegetation succession in dry pastures, which are mosaically intermixed with some oak forests.

## Data Availability

The data presented in this study are available on request from the corresponding author.

## Supplementary Materials

The following supporting information can be downloaded at: https://www.mdpi.com/article/10.3390/plants12193346/s1, Table S1: List of species recorded at investigated localities; Table S2: Results of the Kruskal–Wallis test for equal medians and pairwise Mann–Whitney; for investigated forest types for alpha diversity indices, Ellenberg’s indicator values and measured pH.

## Appendix A. Synoptic Table with Percentage Frequency and Fidelity Index ϕ-Coefficients

**Table A1:** Diagnostic species (f ≥ 50.00; ϕ ≥ 40.00%) are marked in bold.

Forest Type	FA		FB		QA		QB	
Number of Relevés	11		2		4		4	
*Hypnum cupressiforme* Hedw.	100	^44.7^	.		100		50	
*Polytrichum formosum* Hedw.	64		.		100		.	
*Isothecium alopecuroides* (Lam. ex Dubois)	**73**	**^52.6^**	.		25		25	
*Dicranella heteromalla* (Hedw.) Schimp.	64		.		75		.	
*Brachythecium rutabulum* (Hedw.) Schimp.	55		100		25		25	
*Plagiochila porelloides* (Torr. ex Nees)	**73**	**^42.8^**	50		25		.	
*Metzgeria furcata* (L.) Corda	**73**	**^64.9^**	.		25		.	
*Dicranum scoparium* Hedw.	45		.		50		25	
*Hedwigia ciliata* (Hedw.) P.Beauv.	36		50		50		.	
*Pseudanomodon attenuatus* (Hedw.) Ignatov	45		50		25		.	
*Sciuro-hypnum populeum* (Hedw.) Ignatov &	45		50				25	
*Oxyrrhynchium hians* (Hedw.) Loeske	27		100				50	
*Alleniella complanata* (Hedw.) S.Olsson,	36		50		25		.	
*Rhizomnium punctatum* (Hedw.) T.J.Kop.	**55**	**^68.8^**	.				.	
*Thamnobryum alopecurum* (Hedw.) Gangulee	36		100				.	
*Ctenidium molluscum* (Hedw.) Mitt.	27		100				25	
*Plagiothecium cavifolium* (Brid.) Z.Iwats	45		.		25		.	
*Lophocolea heterophylla* (Schrad.) Dumort	45	^62^	.				.	
*Anomodon viticulosus* (Hedw.) Hook. & Tay	27		50				25	
*Pseudotaxiphyllum elegans* (Brid.) Z.Iwat	36		.		25		.	
*Frullania dilatata* (L.) Dumort.	27		.		50		.	
*Radula complanata* (L.) Dumort.	36		.		25		.	
*Atrichum undulatum* (Hedw.) P.Beauv.	27		.		50		.	
*Polytrichum piliferum* Hedw.	9		.		**100**	**^94.3^**	.	
*Atrichum angustatum* (Brid.) Bruch & Schi	27		.		25		.	
*Plagiomnium cuspidatum* (Hedw.) T.J.Kop.	36		.				.	
*Metzgeria conjugata* Lindb.	27		.		25		.	
*Mnium stellare* Hedw.	18		**100**	**^89.2^**			.	
*Plagiomnium rostratum* (Schrad.) T.J.Kop.	18		**100**	**^89.2^**			.	
*Pogonatum urnigerum* (Hedw.) P.Beauv.	27		.		25		.	
*Frullania tamarisci* (L.) Dumort.	27		.		25		.	
*Eurhynchium angustirete* (Broth.) T.J.Kop	27		.		25		.	
*Thuidium delicatulum* (Hedw.) Schimp.	18		.				50	
*Brachytheciastrum velutinum* (Hedw.) Igna	36		.				.	
*Diplophyllum albicans* (L.) Dumort.	27		50				.	
*Plagiomnium undulatum* (Hedw.) T.J.Kop.	18		**100**	**^89.2^**			.	
*Brachythecium tommasinii* (Sendtn. ex Bou)	9		**100**	**^81.3^**	25		.	
*Ptychostomum capillare* (Hedw.) Holyoak &	9		50		25		25	
*Didymodon fallax* (Hedw.) R.H.Zander	.		50		25		50	
*Ceratodon purpureus* (Hedw.) Brid.	.		.		**75**	**^66.7^**	25	
*Leucodon sciuroides* (Hedw.) Schwägr.	.		.		25		**75**	**^66.7^**
*Hylocomium splendens* (Hedw.) Schimp.	27		.				.	
*Pogonatum aloides* (Hedw.) P.Beauv.	27		.				.	
*Solenostoma gracillimum* (Sm.) R.M.Schust	18		.		25		.	
*Plagiothecium nemorale* (Mitt.) A.Jaeger	27		.				.	
*Pseudotaxiphyllum elegans* (Brid.) Z.Iwat	18		.		25		.	
*Pleurozium schreberi* (Willd. ex Brid.) M	18		.		25		.	
*Dicranum fulvum* Hook.	27		.				.	
*Conocephalum salebrosum* Szweyk., Buczk.	9		**100**	**^94.3^**			.	
*Pedinophyllum interruptum*(Nees) Kaal.	18		.		25		.	
*Plagiomnium affine* (Blandow ex Funck) T.	9		50		25		.	
*Pterigynandrum filiforme* Hedw.	27		.		.		.	
*Paraleucobryum longifolium* (Hedw.) Loesk	18		.		.		25	
*Scapania nemorea* (L.) Grolle	27		.		.		.	
*Leucobryum juniperoideum* (Brid.) Müll.Ha	9		.		50		.	
*Blepharostoma trichophyllum* (L.) Dumort.	18		.		25		.	
*Lepidozia reptans* (L.) Dumort.	18		.		25		.	
*Lophozia ventricosa* (Dicks.) Dumort.	18		.		25		.	
*Brachythecium salebrosum* (Hoffm. ex F.We	18		.		.		25	
*Exsertotheca crispa* (Hedw.) S.Olsson, En	9		50		.		25	
*Homalothecium philippeanum* (Spruce) Schi	9		100	^94.3^	.		.	
*Orthotrichum cupulatum* Brid.	9		.		25		25	
*Barbula unguiculata* Hedw.	.		.		.		**75**	**^83.2^**
*Tortula muralis* Hedw.	.		.		.		**75**	**^83.2^**
*Encalypta streptocarpa* Hedw.	.		.		.		**75**	**^83.2^**
*Diplophyllum obtusifolium* (Hook.) Dumort	18		.		.		.	
*Mnium marginatum* (Dicks.) P.Beauv.	18		.		.		.	
*Chiloscyphus polyanthos* (L.) Corda	18		.		.		.	
*Plagiochila asplenioides* (L.) Dumort	18		.		.		.	
*Dichodontium pellucidum* (Hedw.) Schimp.	18		.		.		.	
*Cirriphyllum crassinervium* (Taylor) Loes	18		.		.		.	
*Bartramia pomiformis* Hedw.	18		.		.		.	
*Pohlia nutans* (Hedw.) Lindb.	9		.		25		.	
*Dicranella varia* (Hedw.) Schimp.	9		.		.		25	
*Antitrichia curtipendula* (Hedw.) Brid.	9		.		25		.	
*Plagiothecium denticulatum* (Hedw.) Schim	18		.		.		.	
*Calypogeia fissa* (L.) Raddi	18		.		.		.	
*Pellia neesiana* (Gottsche) Limpr.	9		50		.		.	
*Solenostoma hyalinum* (Lyell) Mitt.	18		.		.		.	
*Cephaloziella divaricata* (Sm.) Schiffn.	9		.		25		.	
*Homalia trichomanioides* (Hedw.) Brid.	18		.		.		.	
*Mnium hornum* Hedw.	18		.		.		.	
*Heterocladium heteropterum* (Brid.) Schim	18		.		.		.	
*Polytrichastrum alpinum* (Hedw.) G.L.Sm.	9		.		25		.	
*Conocephalum conicum* (L.) Dumort.	18		.		.		.	
*Polytrichum juniperinum* Hedw.	9		.		25		.	
*Hylocomiadelphus triquetrus* (Hedw.) Ochy	9		.		.		25	
*Fissidens dubius* P.Beauv.	9		.		.		25	
*Dicranoweisia cirrata* (Hedw.) Lindb.	9		.		25		.	
*Orthotrichum anomalum* Hedw.	9		.		25		.	
*Syzygiella autumnalis* (DC.) K.Feldberg,	9		.		25		.	
Grimmia trichophylla Grev.	9		.		25		.	
*Pseudoleskeella catenulata* (Brid. ex Sch	9		.		25		.	
*Lewinskya striata* (Hedw.) F.Lara, Garill	9		.		25		.	
*Rhabdoweisia fugax* (Hedw.) Bruch. & Schi	9		.		25		.	
*Platygyrium repens* (Brid.) Schimp.	9		.		25		.	
*Orthotrichum stramineum* Hornsch. ex Brid	9		.		25		.	
*Lewinskya speciosa* (Nees) F.Lara, Garill	9		.		25		.	
*Cynodontium polycarpon* (Hedw.) Schimp.	9		.		25		.	
*Schistidium crassipilum* H.H.Blom	9		.		.		25	
*Cephalozia bicuspidata* (L.) Dumort.	.		50		25		.	
*Fissidens taxifolius* Hedw.	.		50		.		25	
*Thuidium recognitum* (Hedw.) Lindb.	.		.		25		25	
*Didymodon rigidulus* Hedw.	.		.		25		25	
*Hypnum cupressiforme* var. *lacunosum* Brid.	.		.		25		25	
*Homalothecium lutescens* (Hedw.) H.Rob.	.		.		.		**50**	**^65.5^**
*Didymodon acutus* (Brid.) K.Saito	.		.		.		**50**	**^65.5^**
*Homalothecium sericeum* (Hedw.) Schimp.	.		.		.		**50**	**^65.5^**
*Grimmia pulvinata* (Hedw.) Sm.	.		.		.		**50**	**^65.5^**
*Tortella tortuosa* (Hedw.) Limpr.	.		.		.		**50**	**^65.5^**
*Flexitrichum flexicaule* (Schwägr.) Ignat	.		.		.		**50**	**^65.5^**
*Plagiothecium platyphyllum* Mönk.	9		.		.		.	
*Leskea polycarpa* Hedw.	9		.		.		.	
*Grimmia hartmanii* Schimp.	9		.		.		.	
*Porella arboris-vitae* (With.) Grolle	9		.		.		.	
*Dicranum spurium* Hedw.	9		.		.		.	
*Diphyscium foliosum* (Hedw.) D.Mohr	9		.		.		.	
*Leucobryum glaucum* (Hedw.) Ĺngstr.	9		.		.		.	
*Jungermannia pumila* With.	9		.		.		.	
*Polytrichum commune* Hedw.	9		.		.		.	
*Sphagnum quinquefarium* (Braithw.) Warnst	9		.		.		.	
*Porella platyphylla* (L.) Pfeiff.	9		.		.		.	
*Pseudoscleropodium purum* (Hedw.) M.Fleis	9		.		.		.	
*Dicranum polysetum* Sw. ex anon.	9		.		.		.	
*Bazzania trilobata* (L.) Gray	9		.		.		.	
*Porella baueri* (Schiffn.) C.E.O.Jensen	9		.		.		.	
*Lewinskya affinis* (Schrad. ex Brid.) F.L	9		.		.		.	
*Callicladium imponens* (Hedw.) Hedenäs, S	9		.		.		.	
*Ulota bruchii* Hornsch. ex Brid.	9		.		.		.	
*Campylopus pyriformis* (Schultz) Brid.	9		.		.		.	
*Plagiothecium succulentum* (Wilson) Lindb	9		.		.		.	
*Plagiothecium laetum* Schimp.	9		.		.		.	
*Microlejeunea ulicina* (Taylor) Steph.	9		.		.		.	
*Amblystegium serpens* (Hedw.) Schimp.	9		.		.		.	
*Tritomaria exsectiformis* (Breidl.) Schif	9		.		.		.	
*Isothecium myosuroides* Brid.	9		.		.		.	
*Sciuro-hipnum flotowianum* (Sendtn.) Ignatov	9		.		.		.	
*Sciuro-hypnum plumosum* (Hedw.) Ignatov &	9		.		.		.	
*Taxiphyllum wissgrillii* (Garov.) Wijk &	9		.		.		.	
*Pellia epiphylla* (L.) Corda	.		50		.		.	
*Plasteurhynchium striatulum* (Spruce) M.F	.		50		.		.	
*Rhynchostegiella teneriffae* (Mont.) Dirk	.		50		.		.	
*Gymnostomum calcareum* Nees & Hornsch.	.		50		.		.	
*Liochlaena lanceolata* Nees	.		50		.		.	
*Conardia compacta* (Drumm. ex Müll.Hal.) H	.		50		.		.	
*Seligeria pusilla* (Hedw.) Bruch & Schimp	.		50		.		.	
*Plagiomnium ellipticum* (Brid.) T.J.Kop.	.		50		.		.	
*Barbilophozia barbata* (Schmidel ex Schre	.		.		25		.	
*Funaria hygrometrica* Hedw.	.		.		25		.	
*Orthotrichum pallens* Bruch ex Brid.	.		.		25		.	
*Amphidium mougeotii* (Schimp.) Schimp.	.		.		25		.	
*Grimmia muehlenbeckii* Schimp.	.		.		25		.	
*Ulota crispula* Bruch	.		.		25		.	
*Abietinella abietina* (Hedw.) M.Fleisch.	.		.		.		25	
*Ptychostomum rubens* (Mitt.) Holyoak & N	.		.		.		25	
*Ptychostomum moravicum* (Podp.) Ros & Maz	.		.		.		25	
*Syntrichia montana* Nees	.		.		.		25	
*Trichostomum brachydontium* Bruch	.		.		.		25	
*Tortella inclinata* (R.Hedw.) Limpr.	.		.		.		25	
*Thuidium assimile* (Mitt.) A.Jaeger	.		.		.		25	
*Encalypta vulgaris* Hedw.	.		.		.		25	
*Didymodon vinealis* (Brid.) R.H.Zander	.		.		.		25	
*Tortula lindbergii* Broth.	.		.		.		25	
*Weisia longifolia* Mitt.	.		.		.		25	
*Homomallium incurvatum* (Schrad. ex Brid.	.		.		.		25	
*Bryum argenteum* Hedw.	.		.		.		25	
*Brachythecium glaerosum* (Bruch ex Spruce	.		.		.		25	
*Campyliadelphus chrysophyllus* (Brid.) R.	.		.		.		25	
*Bryum ruderale* Crundw. & Nyholm	.		.		.		25	
*Weisia controversa* Hedw.	.		.		.		25	
*Didymodon spadiceus* (Mitt.) Limpr.	.		.		.		25	
*Syntrichia ruralis (Hedw.)* F.Weber & D.M	.		.		.		25	
*Tortella fasciculata* (Culm.) Culm.	.		.		.		25	
*Syntrichia calcicola* J.J.Amann	.		.		.		25	
*Tortella squarrosa* (Brid.) Limpr.	.		.		.		25	
*Bryoerythrophyllum recurvirostrum* (Hedw.)	.		.		.		25	
*Didymodon sinuosus* (Mitt.) Delonge	.		.		.		25	
*Pulvigera lyellii* (Hook. & Taylor) Pláše	.		.		.		25	
*Dicranella howei* Renauld & Cardot	.		.		.		25	
*Trichostomum crispulum* Bruch	.		.		.		25	
*Campylophyllopsis calcarea* (Crundw. & Ny)	.		.		.		25	
*Streblotrichum convolutum* (Hedw.) P.Beau	.		.		.		25	
*Didymodon cordatus* Jur.	.		.		.		25	
*Weissia brachycarpa* (Nees et Hornsch.) J	.		.		.		25	

## Appendix B. Investigated Localities

**Table A2:** List of investigated localities and habitat characteristics.

Locality		Coordinates WGS84		Habitat Characteristics				
No.	Name	y	x	Geological Bedrock	Forest Type	Vegetation	pH (H_2_O)	pH (KCl)
1.	Šimića put—Molišće	45.48704	17.64151	quartzite	QA	*Festuco drymeiae-Quercetum petrae*	4.05	3.46
2.	Vrhovački mlin	45.46325	17.57752	sandstone interlayed with algal limestone	QB	*Fraxino orni-Quercetum pubescentis*	8.80	7.97
3.	Vranovo	45.48917	17.56697	phyllite	FA	*Luzulo luzuloidis-Fagenion*	5.32	3.95
4.	Čarugin kamen	45.51306	17.53556	migmatite	FA	*Luzulo luzuloidis-Fagenion*	4.67	4.13
5.	Točak (below peak)	45.57584	17.53478	amphibolite	FA	*Luzulo luzuloidis-Fagenion*, with several *Qurecus petrea* trees	4.30	3.94
6.	Dva hrasta	45.56671	17.50290	migmatite	FA	*Festuco drymeiae-Abietetum*, with only scattered low *Abies alba* trees	4.93	4.02
7.	Rupnica	45.60513	17.53190	albitic rhyolite	QB	*Lathyro nigri-Quercetum petraeae*	6.54	5.91
8.	Gudnoga	45.57435	17.63108	granite	FA	*Luzulo luzuloidis-Fagenion*	4.42	3.95
9.	Kovačica brook, near Jankovac	45.53149	17.69876	gneiss	FA	*Luzulo luzuloidis-Fagenion*	5.36	5.07
10.	Mala Rijeka	45.46467	17.86223	phyllite	FA	*Luzulo luzuloidis-Fagenion*	4.17	3.82
11.	Remetska Rijeka	45.47461	17.86411	chlorite schists	FA	*Luzulo luzuloidis-Fagenion*	4.68	3.79
12.	Djedov nos	45.49369	17.86581	dolomitic breccia-conglomerates	QB	*Lathyro nigri-Quercetum petraeae*	7.78	7.40
13.	Šaševa—Rastova kosa	45.49537	17.81560	slate, quartzite	QA	*Qurecus petraea*-*Calluna vulgaris* community	3.89	3.13
14.	Viljevačka kosa	45.51000	17.80319	schist	QA	*Festuco drymeiae-Quercetum petrae*	4.34	3.47
15.	Pištanska kosa	45.51340	17.76675	sand with grains of schits and phyllites	QA	*Molinio arundinaceae-Quercetum petraeae*	4.79	3.98
16.	Jankovac, near brook spring	45.51867	17.68675	limestone, tuffa	FB	*Vicio oroboidi-Fagetum sylvaticae*	8.48	8.01
17.	Jankovac	45.52304	17.68300	limestone, tuffa	FB	*Vicio oroboidi-Fagetum sylvaticae*	8.12	8.04
18.	Svinjarevac	45.50098	17.52846	quartzite, gneiss	FA		4.27	3.32
19.	Radovanovačke Sokoline	45.47723	17.62205	quartzite	FA	*Luzulo luzuloidis-Fagenion*, with *Quercus cerris* and *Q. petera*	3.93	3.49
20.	Velika Pliš	45.46956	17.64227	dolomites	QB	*Lathyro nigri-Quercetum petraeae* with patches of *Fraxino orni-Quercetum pubescentis*	7.34	7.23
21.	Velika Raduča, Crni virovi	45.53005	17.72536	slate, quartzite	FA	*Luzulo luzuloidis-Fagenion*	4.70	4.13
